# Breast silicone implants’ pericapsular impairment: current underdiagnosed status

**DOI:** 10.3389/fsurg.2023.1249078

**Published:** 2023-09-12

**Authors:** Eduardo de Faria Castro Fleury

**Affiliations:** ^1^ Department of Radiology, Centro Universitário São Camilo—Curso de Medicina, São Paulo, Brazil; ^2^ IBCC Oncologia, São Paulo, Brazil

**Keywords:** breast implant, granuloma, BIA-ALCL, BIA-SCC, breast cancer

## Abstract

Many complications related to silicone implants have been reported recently, from clinical symptoms manifestations to association with some specific types of cancer. During the early 2010s, it was believed that implants were biocompatible and inert to the human body and that gel bleeding/leakage events were rare and without repercussions for the human body. However, at the end of 2010s, several studies pointed out that gel bleeding was more frequent than previously believed, and the pathogenic potential of free silicone should not be ignored. The Food and Drug Administration recommends performing magnetic resonance imaging in asymptomatic patients 5–6 years after implant placement. The descriptors in the Breast Imaging and Reporting Data System lexicon seem outdated for classifying the new generations of implants with cohesive gel, which hinders the diagnosis of device complications. In this review, supported by our research data publications related to silicone implants for 6 years on a prospective study protocol, most of them being original articles, we summarized the main complications observed in clinical practice and discuss the impact of these changes on patients’ outcomes focusing on the pericapsular space.

## 1. Introduction

Many complications related to silicone implants have been reported recently, from clinical symptoms manifestations to association with some specific types of cancer, such as breast implant–associated anaplastic large cell lymphoma (BIA-ALCL) and breast implant–associated squamous cell carcinoma (BIA-SCC), both recognized by the Food and Drug Administration (FDA) agency (1–4).

Since the end of breast implants moratorium at the beginning of the 21st century, the number of surgeries using silicone implants has increased exponentially. Patients opt for these devices for augmentation surgery for esthetic purposes or reconstructive surgeries for breast cancer treatment (5).

During the early 2010s, it was believed that implants were biocompatible and inert to the human body and that gel bleeding/leakage events were rare and without repercussions for the human body (6).

However, at the end of 2010s, several studies pointed out that gel bleeding was more frequent than previously believed and the pathogenic potential of free silicone should not be ignored (7, 8). At the same time, groups of women gathered on social networks associating their morbidities with the presence of breast silicone implants and called the disease as breast implant illness (BII) (9–11). Many recently published manuscripts link the pathogenic potential of silicone with the chronic inflammation promoted by the foreign body (12, 13). One of the main complaints of patients with clinical symptoms related to silicone implants is the difficulty in diagnosing the disease.

The FDA recommends performing magnetic resonance imaging (MRI) in asymptomatic patients 5–6 years after implant placement and an ultrasound scan for patients referred for diagnostic tests (14). Nevertheless, imaging exams follow the recommendations proposed by the American College of Radiology (ACR), incorporated in the Breast Imaging and Reporting Data System (BI-RADS) lexicon 5th edition in a specific chapter. The descriptors in the BI-RADS lexicon seem outdated for classifying the new generation of implants with cohesive gel, which hinders the diagnosis of device complications (15).

The lexicon is practically limited to evaluating the integrity of silicone implants, extracapsular ruptures, and intracapsular collection. The lexicon does not present guidelines for evaluating the pericapsular tissue nor lists the possible descriptors compatible with cohesive gel complications (15).

In this review, supported by our research data publications related to silicone implants for 6 years on a prospective study protocol, most of them being original articles, we summarized the main complications observed in clinical practice and discuss the impact of these changes on patients’ outcomes focusing on the pericapsular space.

## 2. Silicone implants composition

Silicone implants are medical devices approved by the FDA for medical use and included in category III devices. The FDA describes category III as “usually sustain or support life, are implanted or present a potential unreasonable risk of illness or injury” (16).

The implants are composed of polydimethylsiloxane polymers in long and short chains. The most stiff state is in the implant shell and the softest in the inner gel. The latest generation of implants consists of cohesive gels that determine greater product stability. After implant placement, a fibrous capsule forms around it to protect the host from the foreign body. The fibrous capsule is composed of defense cells, including macrophages and lymphocytes (17–19).

Over time, there is degradation on the implant's surface, which determines changes in the shell permeability that allows leakage of the internal content. Studies demonstrate that shorter-chain silicones have more potential for leakage and distant migration. A 2020 study indicates the presence of silicone inside the fibrous capsule and in the pericapsular tissue in 98.8% and 86.6%, respectively. The study also divided the patients into two groups, one with cohesive gel and the other with other types of gel. The study results showed no statistically significant difference for extravasation between groups (20).

When in contact with the fibrous capsule, the silicone particles arising from the degradation/leakage of the device activate the macrophage–antigen complex (MAC). MAC activation will recruit T lymphocytes against the foreign body. Silicone particles in this situation appear in two patterns. The first is extracellular silicone, where giant cells formed around the silicone, associated with intense fibrosis, resulting in a silicone-induced granuloma, described as silicone-induced granuloma of breast implant capsule (SIGBIC). In the second form, which is more related to acute conditions, the silicone phagocytosis by the macrophage is frustrated and results in the macrophage's apoptosis. This process determines the deregulation and perpetuation of the immune response with excessive consumption of lymphocytes. In these cases, the inflammatory process produces exudate (21). Based on our observations, we linked the exudate resulting from the dysregulated inflammatory process with the intracapsular formation of late seroma and hematoma. Lymphocyte consumption may be the reason for the statement by Keane et al. (22) published in a review: “Curiously, a relative attenuation of circulating T-helper cells may occur in the first couple days following placement of a textured, but not smooth breast implant. While each of these studies proposes a different ‘trigger’, chronic inflammation is the common thread and is the most likely facilitator of malignant transformation to BIA-ALCL.” Despite the original article demonstrating more significant postoperative leukopenia in patients with textured implants when compared with smooth implants (23), we published a case report of BIA-ALCL in a patient who had SIGBIC and signs of gel bleeding in both breasts, with leukopenia in preoperative exams. In the postoperative period of the en bloc capsulectomy, there was normalization of leukocyte levels in peripheral blood (24).

## 3. Silicone implant location

According to the BI-RADS lexicon, the MRI may report two implant locations. The first is retroglandular placement, where the implant is anterior to the pectoralis muscle, and second is retropectoral placement, deep into the pectoralis muscle (15). The site for the implant placement decision is shared between the surgeon and the patient. Implant locations have advantages and disadvantages that must be evaluated before surgery. Retroglandular surgery is less complicated, with fewer postoperative complications but less satisfactory esthetic results. Conversely, retropectoral placement has a more complex surgical time, with postoperative complications and a more satisfactory esthetic result. The retropectoral site is recommended for breast cancer screening because it has a smaller area of the penumbra (overlap of the implant over the breast parenchyma) than the retroglandular site (15, 25, 26).

## 4. Association of breast implants with neoplasms

The association between silicone implants and breast neoplasms is quite controversial. Few studies in the literature discuss the topic. The FDA recognizes two implant-related malignancies, BIA-ALCL and BIA-SCC. However, numerous case reports linking implants with neoplasms are found in digital medical libraries, such as angiosarcoma. There are also reports of fibromatosis originating from the silicone implant placement. Studies in the literature converge on the difficulty of determining the triggering factor for these neoplasms’ development (27–29).

In a 2021 study, our group published a theory that chronic and persistent inflammation resulting from breast implant placement could be the main factor in determining metaplasia and dysplasia of cells exposed to the chronic inflammatory process (30). We also published a case report that shows evidence of areas of silicone leakage with the appearance of breast neoplasms, with a benign papillary lesion diagnosis (31). The studied tumor's origins were closely linked to the areas of exposure to the silicone gel. Tumors arising from an inflammatory process are nothing new in medicine. This category includes cervix cancer and human papillomatosis virus (HPV), basal cell cancer and sun exposure, esophageal cancer from chronic reflux, and Barrett's metaplasia (30).

## 5. Diagnostic methods for silicone implants

The FDA recommends MRI as the imaging modality of choice for diagnosing and screening implant-related complications (16). Ultrasonography can be an alternative due to its wide availability, but its low reproducibility is the main limiting factor.

The BI-RADS lexicon used to describe changes in the breast has a specific chapter for evaluating implants. The lexicon is restricted to classifying implants according to type, location, intracapsular rupture diagnosis, and extracapsular silicone presence. The lexicon was launched in 2013 when non-cohesive gel implants were prevalent (15). Descriptors related to the cohesive gel are not described by the lexicon, which makes their descriptors outdated.

Since 2017, our group has started a research protocol to evaluate silicone implants. Among the findings described, the ability of magnetic resonance imaging to detect gel bleeding stands out. The finding of gel bleeding on MRI was described as SIGBIC. SIGBIC is a gel bleeding marker, whose descriptor is “intracapsular heterogeneous tissue with late contrast enhancement compatible to silicone-induced granuloma” (32).

**Figure 1:**
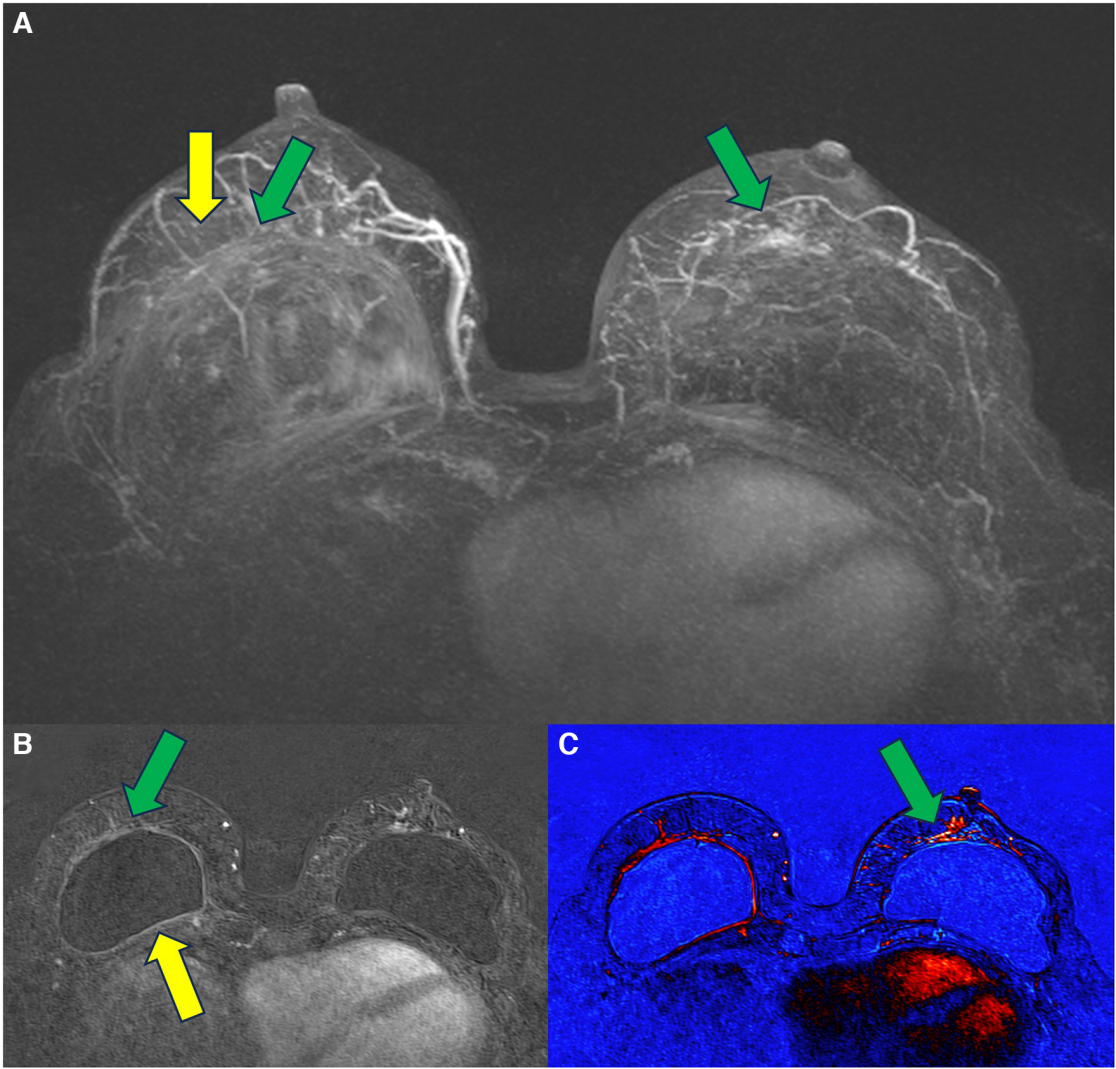
A 58-year-old woman with silicone implants for 6 years, with suspicion of right capsular contracture. The multiplanar intensity projection (MIP) reconstruction (**A**), the axial post-contrast image (**B**), and the flow intensity image (**C**) show capsular contracture with inflammatory signs in the right breast (yellow arrows). The green arrows show non-mass enhancement of the pericapsular tissue, especially in the right breast. The right implant is an example of fibrous capsule grade 4 and the left as a grade 3.

In 2021, we published a magnetic resonance classification proposal for fibrous capsules, divided into four evolutionary categories, with category 4 representing changes indicative of a pericapsular inflammatory process. The classification graduates the degree of fibrous capsule impairment and the association with an acute inflammatory process (33) (Figure 1).

## 6. The pericapsular space

**Figure 2:**
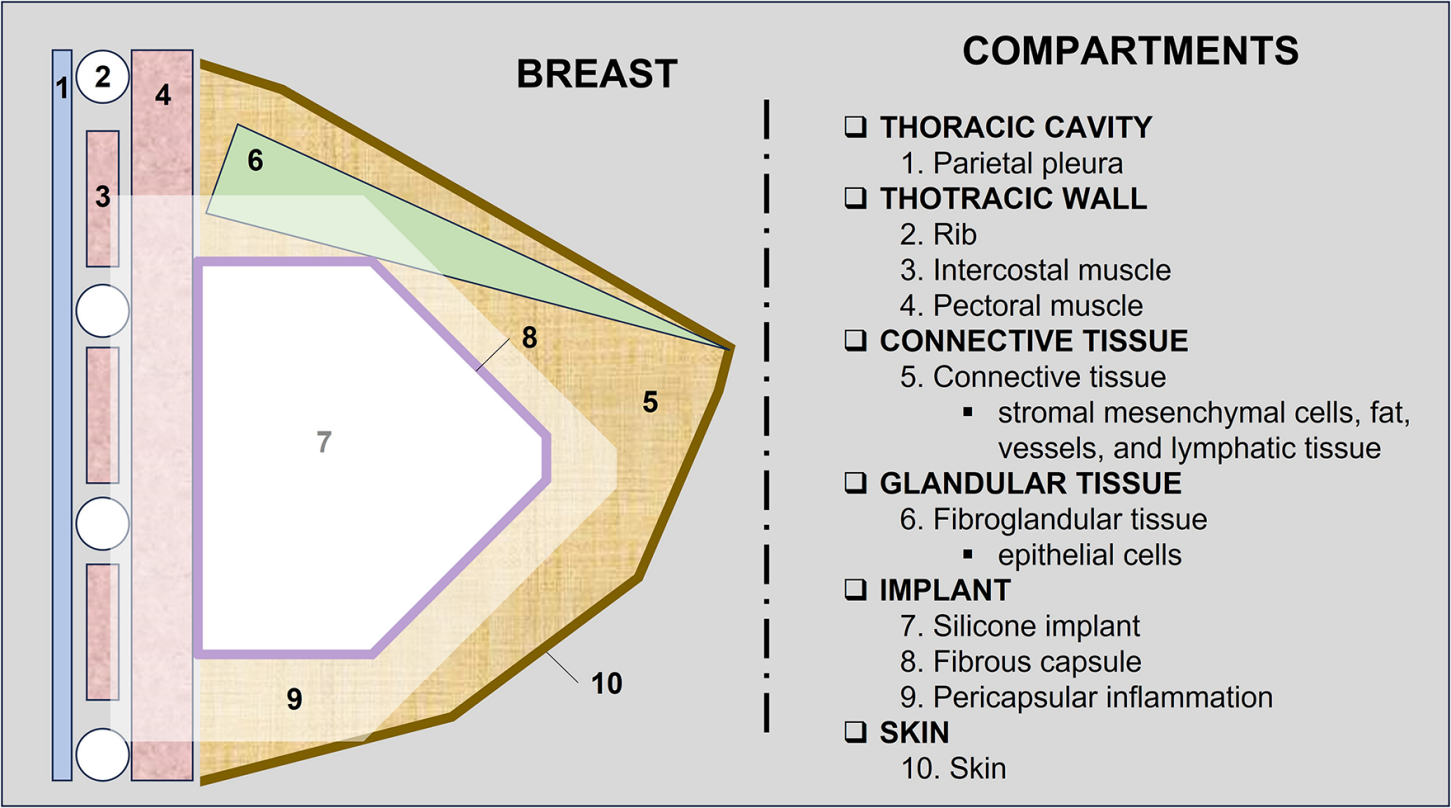
Illustration of breast compartments and structures to be evaluated in breast MRI.

The pericapsular space can be divided according to the structures that compose it. In a didactic way, we divided the pericapsular space into (1) thoracic wall, (2) connective tissue, (3) fibroglandular tissue, and (4) skin (Figure 2).

### 6.1. Chest wall

**Figure 3:**
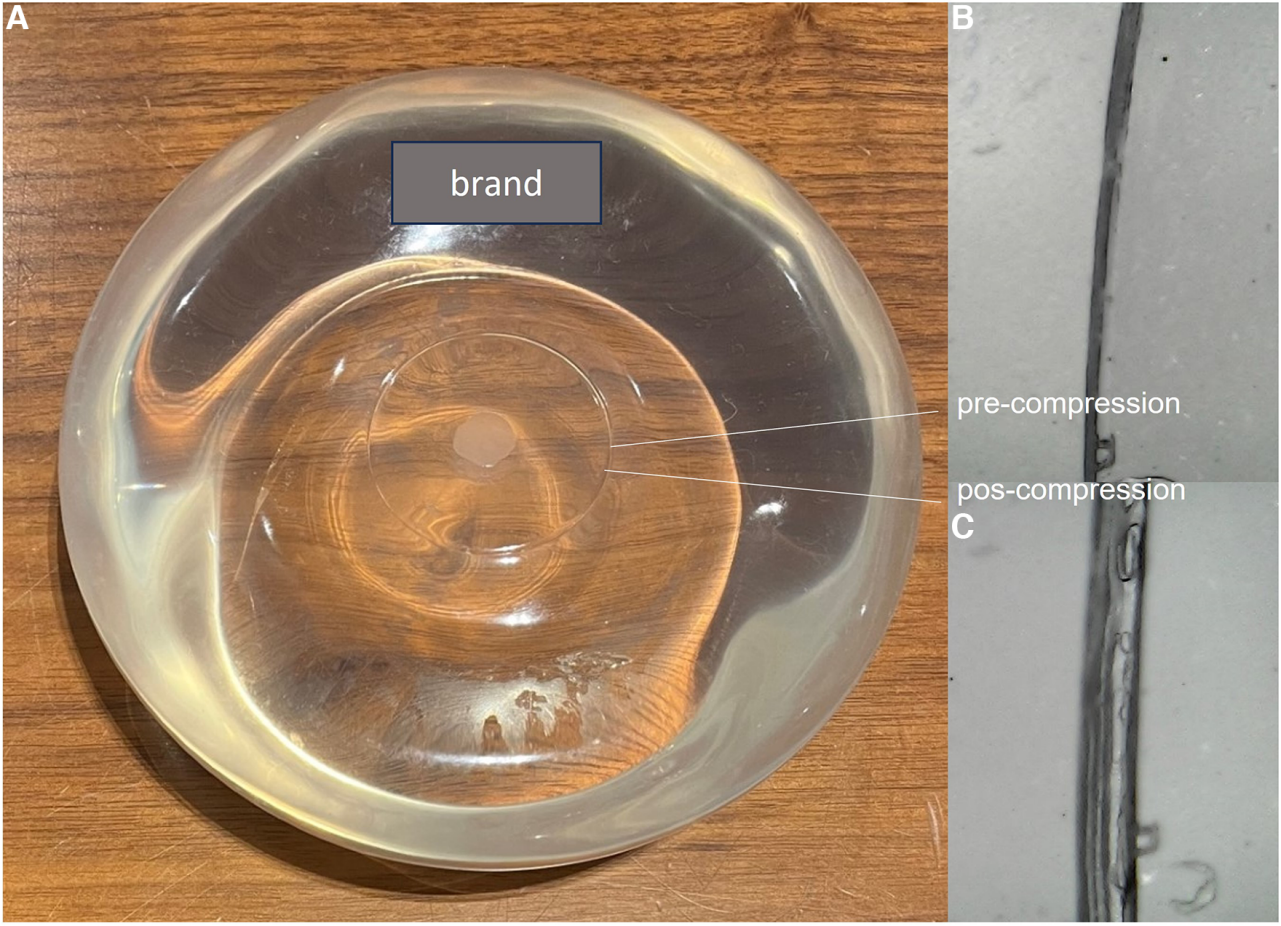
Apparent intact smooth silicone implant (**A**). The posterior seal before (**B**) and after (**C**) compression shows discontinuity of the seal with exposure to the internal cohesive gel content (**C**).

The chest wall will usually meet the posterior surface of the silicone implants. In this topography, implant closure seals are usually found and serve as a reference for diagnosing rotation. The closing seal is important because it is the area of most significant silicone leakage, with direct exposure of the silicone to the fibrous capsule (Figure 3). In a review by Keane et al. (22), the authors stated intraoperative findings of BIA-SCC that include fungating breast capsule masses with granulomatous and keratinized debris contained within a viscous, turbid seroma fluid. The article also discussed that the malignancy arises from the posterior aspect of the implant capsule. Curiously, the tumor grew in the same topography reported in our manuscript, where we describe a patient that developed breast carcinoma at the site of silicone exposure in the closing seal defect (30).

In the retroglandular plane, the fibrous capsule is in close contact with the pectoral muscle. Chronic inflammation of the fibrous capsule can cause contiguity to invade the pectoral muscle, making it difficult to perform an en bloc capsulectomy. Sometimes it is required to remove the superficial fibers of the compromised muscle with the diseased fibrous capsule.

**Figure 4:**
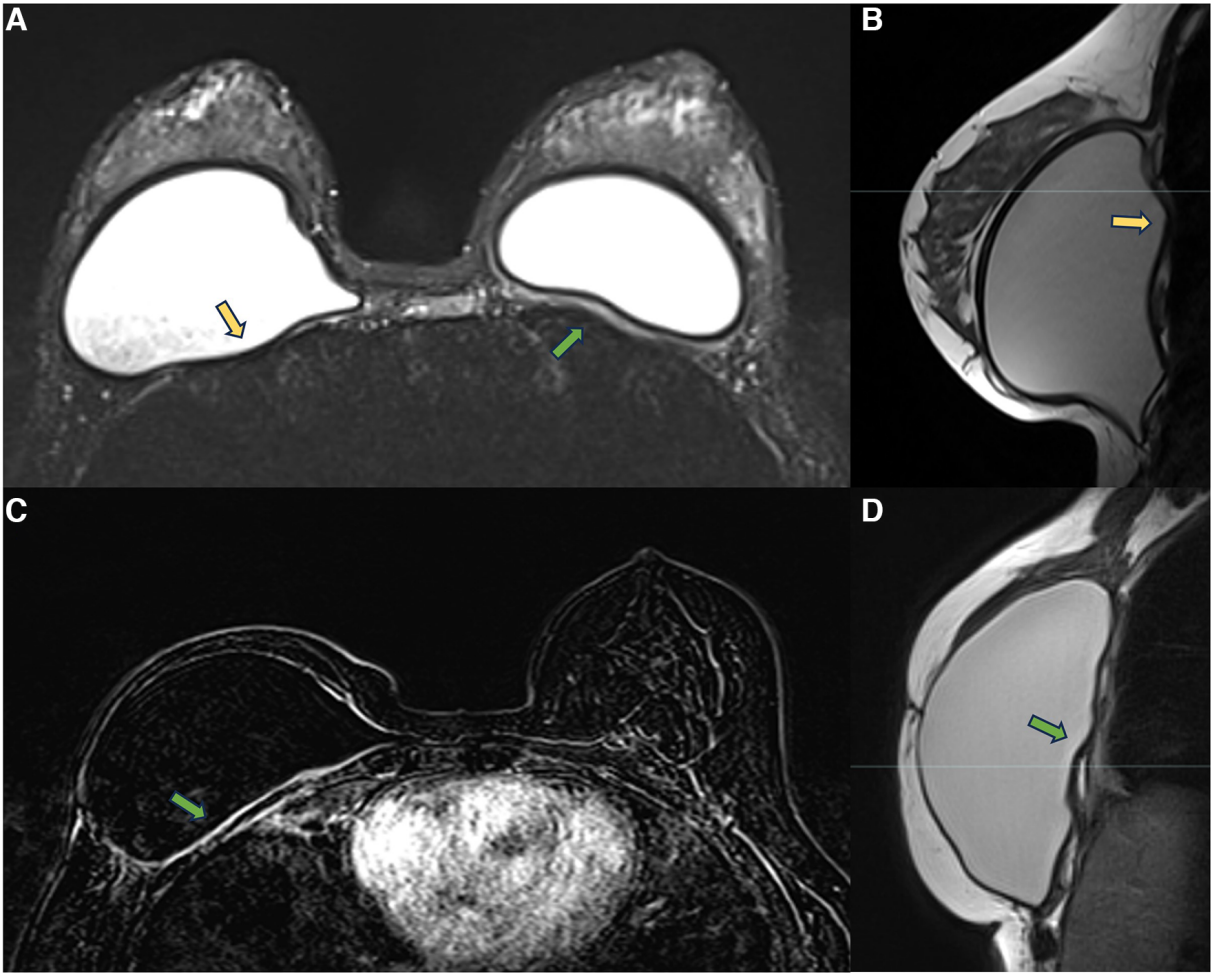
A 28-year-old woman with esthetic retropectoral silicone for 4 years (**A,B**), and a 55-year-old woman with a reconstructive surgery of the right breast for   years. Axial T2** image (**A**) and sagittal proton density image (**B**) show a subpectoral bilateral implant. The yellow arrows show the herniation of the implant surface to the intercostal space. Atrophy of the intercostal muscle and thickening of the fibrous capsule in contact with the parietal pleura are observed. Axial post-contrast image (**C**) and sagittal proton density image (**D**) show capsular contracture associated with pericapsular edema. The extension of the enhancement to the parietal pleura is also observed (green arrow).

In the retropectoral plane, the implant is located between the pectoral muscle and the chest wall. The device promotes pectoral muscle stretching and compresses the chest wall. Over time, the pectoral muscle becomes atrophic, as does the intercostal musculature. These muscles’ atrophy favors implant herniation into the intercostal spaces. In this context, the combination of a thickened capsule, implant herniation into the intercostal space, and atrophy of the intercostal muscles result in proximity between the fibrous capsule and the thoracic cavity, sometimes encountering the parietal pleura. These processes related to thoracic muscle atrophy and the presence of a foreign body may hinder thoracic expansion (Figure 4). Many patients report significant improvement in respiratory capacity in the immediate postoperative period of explant surgery.

Measuring the distance between the fibrous capsule and the parietal pleura in retropectoral implants may be necessary for the surgical programming of implant removal to minimize intraoperative complications. We generally value measurements with distances smaller than 0.5 cm.

**Figure 5:**
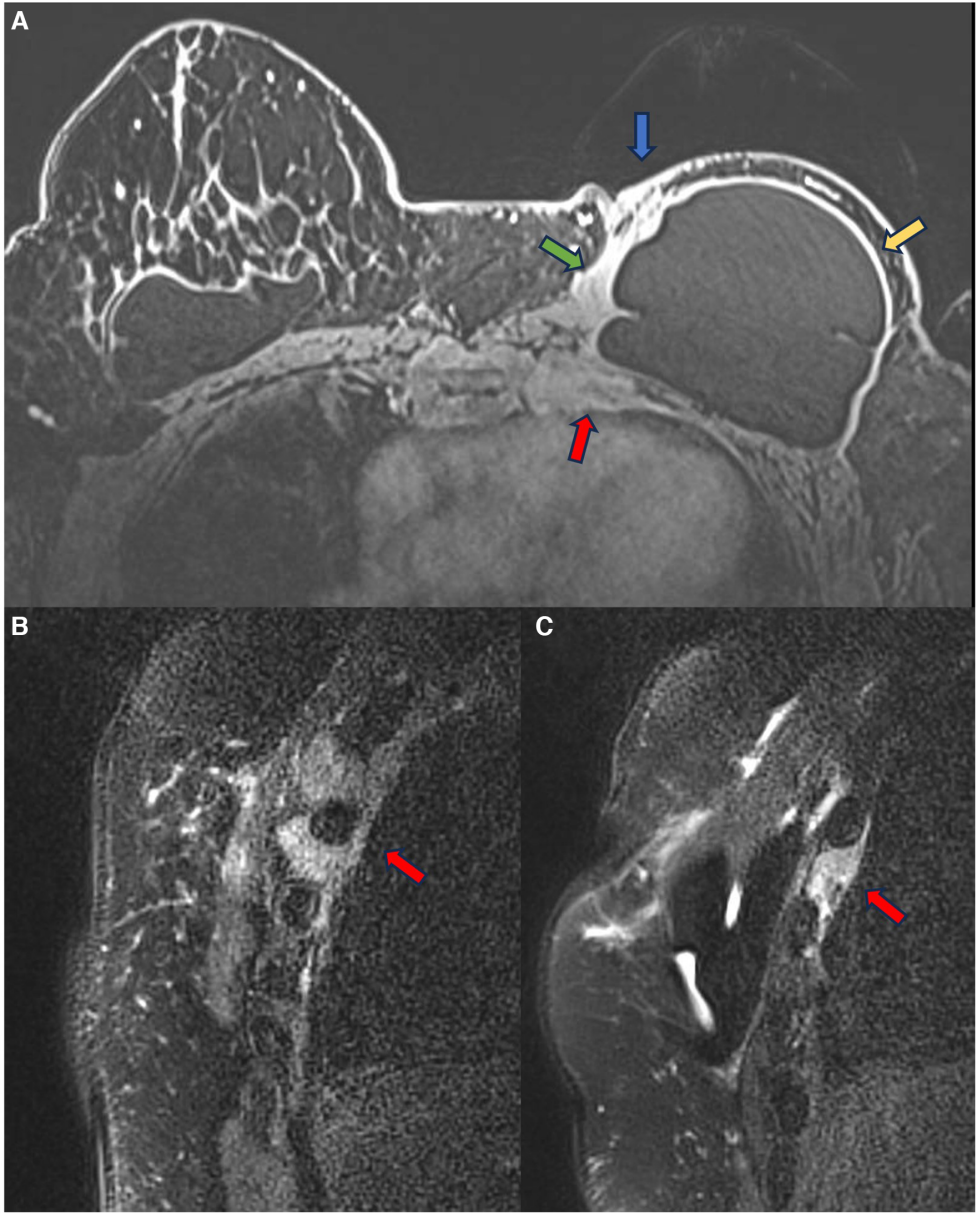
A 48-year-old woman with reconstructive retropectoral silicone for 6 years. Axial T1 post-contrast image (**A**) and sagittal T1 post-contrast images at different sites (**B,C**) show a left capsular contracture associated with edema (yellow arrow). There is contiguity of the fibrous capsule to the pectoral muscle (green arrow) and the skin (blue arrow). The inflammatory process also extends to the thoracic wall, involving the ribs and intercostal muscles.

In episodes of recurrent/remitting acute inflammation, the established chronic inflammatory process can extend to the thoracic wall structures, involving the ribs, intercostal musculature, and costosternal joints. Magnetic resonance imaging is the best method to assess the involvement of the chest wall and allows for distinguishing the compromised structures by the inflammatory process (Figure 5). The differential diagnosis in this situation is malignant neoplasia, especially sarcomas and desmoid tumors, thus having the convenience to continue the clinical investigation in the presence of a pericapsular inflammatory process of the chest wall (27, 28).

### 6.2. Connective tissue

The connective tissue of the breasts is formed essentially by fat, especially over the years when fibroglandular tissue is replaced by adipose tissue. Mesenchymal cells and fibroblasts, blood vessels, lymphatic system, and nervous system cells are also present.

#### 6.2.1. Steatonecrosis

Acute inflammation of the fibrous capsule usually determines edema, architectural distortions, and atypical enhancement of the pericapsular tissue on magnetic resonance imaging. In these cases, it is possible to observe the thickening and enhancement of the fibrous capsule compatible with capsular contracture associated with the pericapsular tissue impairment. The degree of impairment and extension will depend on the intensity of the inflammatory process. Steatonecrosis is most frequently associated with post-surgical complications of silicone implants. The clinical complaints are variable and depend on the tissue involvement extension (34, 35).

**Figure 6:**
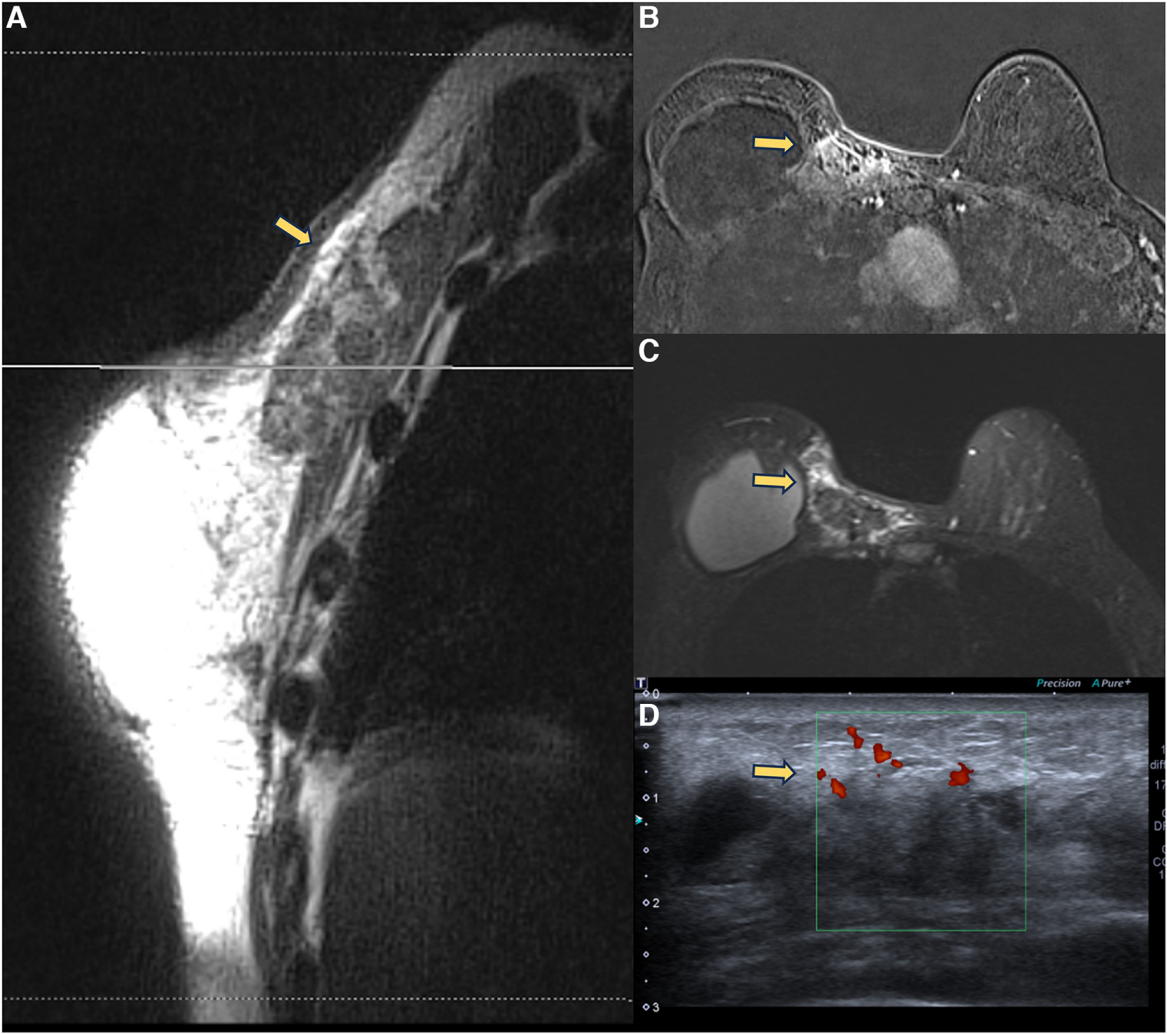
A 71-year-old woman with breast reconstructive surgery with implants for 5 years, presenting with a palpable mass in the medial quadrant. Sagittal proton density image (**A**), axial post-contrast imaging (**B**), axial T2** sequence (**C**), and ultrasonography show a heterogeneous mass with fat content and moderate enhancement, associated with skin thickening and without cleavage plan with the pectoral muscle. The ultrasound (**D**) shows a vascularized heterogeneous mass. The biopsy diagnosis was steatonecrosis.

Steatonecrosis often manifests as palpable, hardened masses, which may be associated with breast edema. Pericapsular steatonecrosis with acute inflammatory signs should be categorized as BI-RADS 4 because they are new and developing (Figure 6).

#### 6.2.2. BIA-ALCL and BIA-DLBCL

The first neoplasm recognized as originating from silicone implants was BIA-ALCL. BIA-ALCL expresses monoclonal proliferation of T lymphocytes, with CD30-positive and ALK-negative markers. ALK is a lymphoma marker, and, as it is negative, there are questions regarding the classification of BIA-ALCL as a true lymphoma (2). In the literature, there are reports of patients with spontaneous remission of BIA-ALCL from diagnosis to surgical excision without specific treatment (36).

Compromise of the pericapsular space in cases of BIA-ALCL is uncommon. According to the 2019 NCCN Consensus Guidelines on the Diagnosis and Treatment of BIA-ALCL, when the lesion invades the pericapsular space, it should be considered as stage 4 according to the TNM criteria (37). In our experience, we had a case of spontaneous remission of extracapsular involvement in a patient who refused to undergo adjuvant chemotherapy after BIA-ALCL diagnosis in explant surgery. In the follow-up exams, there was spontaneous remission of the residual lesion by PET-CT in a 3-year interval.

**Figure 7:**
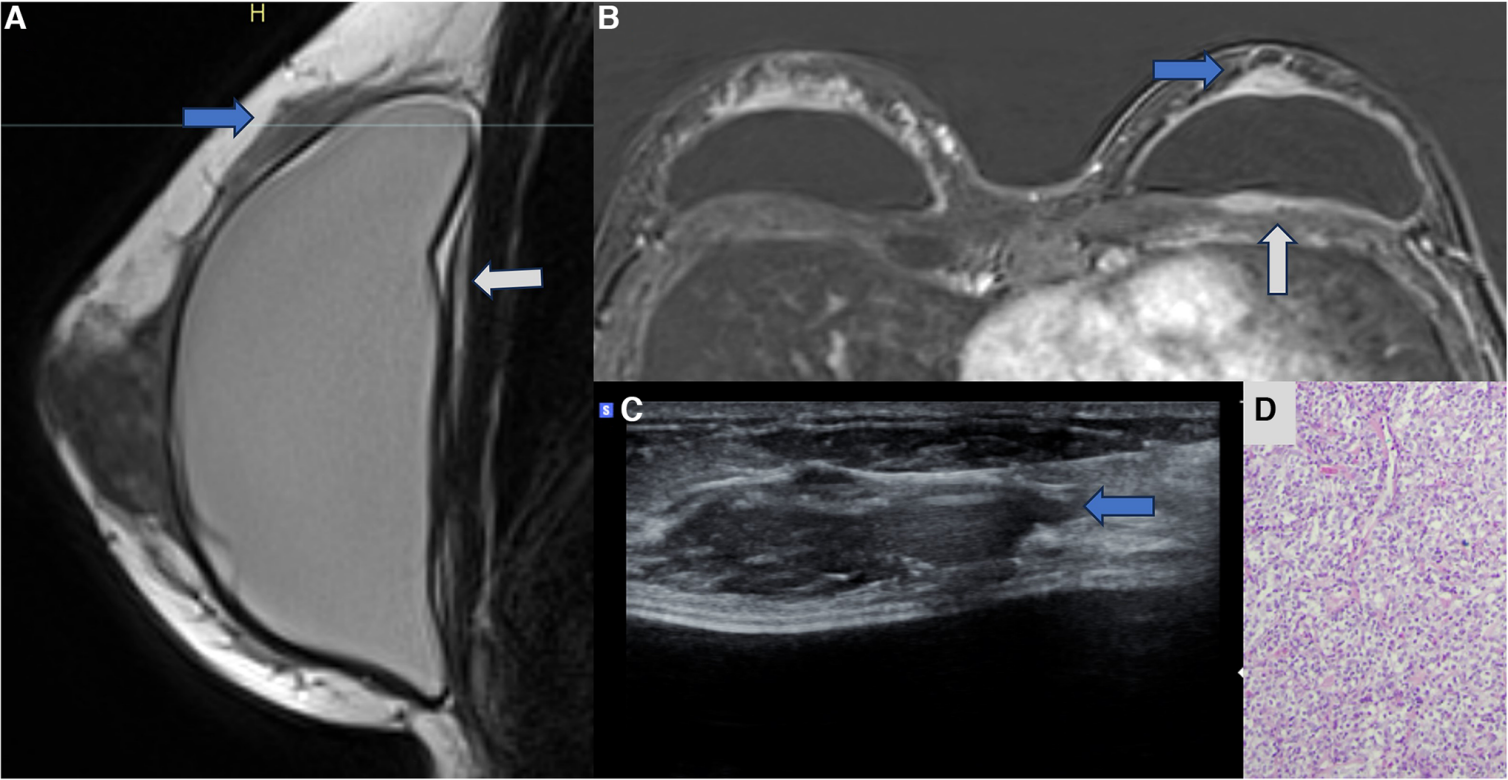
A 47-year-old woman with a palpable lump in the right breast. The sagittal proton density image (**A**), axial post-contrast MRI image (**B**), and ultrasonography (**C**) show a pericapsular mass in contact with the fibrous capsule, with moderate enhancement (blue arrows). There is also capsular contracture and SIGBIC (white arrow). The biopsy specimen (**D**) shows atypical lymphoid proliferation immunophenotype B.

Recently, some cases of breast implant–associated diffuse large B-cell lymphoma (BIA-DLBCL) have been reported in the medical literature. BIA-DLBCL is composed of cellular heterogeneity where the presence of giant B cells stands out. Some studies associate BIA-DLBCL with Epstein–Barr virus (38–40). Magnetic resonance imaging is the method of choice for diagnosing BIA-ALCL and BIA-DLBCL, and it is worth noting that any new lesion compromising the pericapsular environment and presenting contrast enhancement should be considered suspicious. The enhancement of these lesions indicates cellular metabolic activity (Figure 7).

#### 6.2.3. Sarcoma

Sarcoma is a type of spindle cell lesion, extremely rare, accounting for less than 1% of breast cancers. The cause of sarcomas is not well established, and angiosarcoma is the primary type described. There are also reports of fibrosarcoma associated with free silicone in the breast. The differential diagnosis of spindle cell lesions is challenging because it is found in benign, malignant, and reactional lesions. The clinical history contributes to the differential diagnosis. When the cells present atypia, the differential diagnoses are spindle cell metaplastic carcinoma, adenomyoepithelioma, adenosarcoma, osteosarcoma, and myofibroblastic sarcoma. In the absence of atypia, fibromatosis, granulation tissue, pseudoangiomatous stromal hyperplasia (PASH), low-grade adenosquamous carcinoma of the breast, myofibroblastoma, inflammatory myofibroblastic tumor, nodule with spindle cells, lipoma with spindle cells, schwannoma, and neurofibromas should be considered as differentials (41–43).

**Figure 8:**
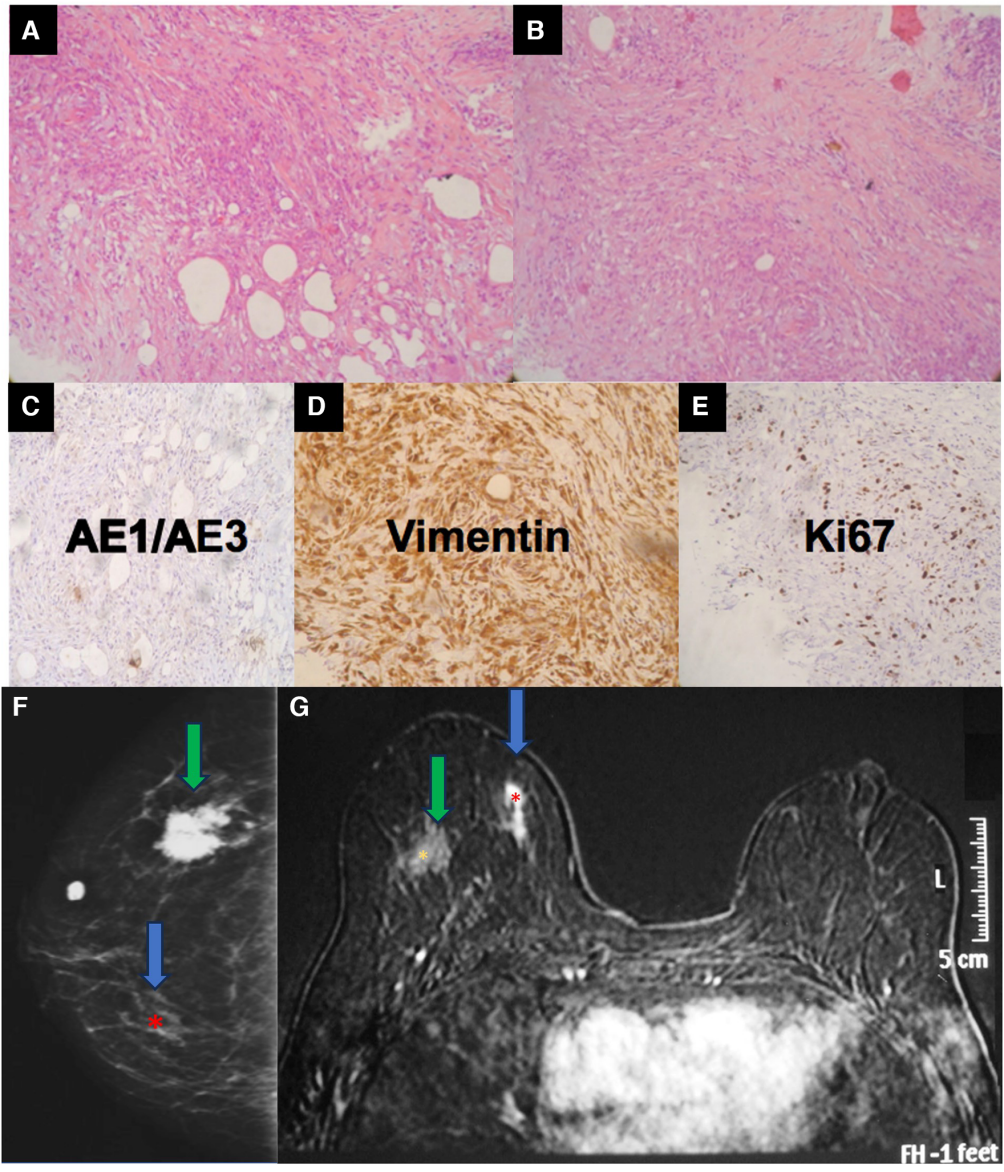
A 34-year-old woman with a history of industrial silicone injection for bilateral breast augmentation. (**A,B**) show the tumor composed of the spindle to epithelioid cells with a fibrohistiocytic appearance. The center is composed of more spindle cells. The tumor shows a negative reaction to AE1/AE3 (**C**), a strong reaction to vimentin (**D**), and an intermediate expression of KI-67 (**E**). Vimentin confirms the mesenchymal origin of the tumor. The mammography (**F**) shows a siliconoma (green arrow) as a spiculated mass and an architectural distortion in the site of the tumor (blue arrow). The MRI shows the early enhancement of the spindle cell tumor (blue arrow) (**G**).

Magnetic resonance imaging is the most indicated imaging method to assess connective tissue impairment in patients with complications related to silicone implants. As in cases of chest wall involvement, these lesions should follow a diagnostic investigation to rule out malignancy (Figure 8).

### 6.3. Glandular tissue

The diagnosis of epithelial lesions related to silicone implants is hampered by the high prevalence of these lesions in the general population. The current knowledge is that silicone implants do not increase the risk of breast carcinomas. However, some case reports and theories in the literature associate the onset of cancer with silicone implants. Thus, we need more scientific evidence to support this association (30, 44–46).

**Figure 9:**
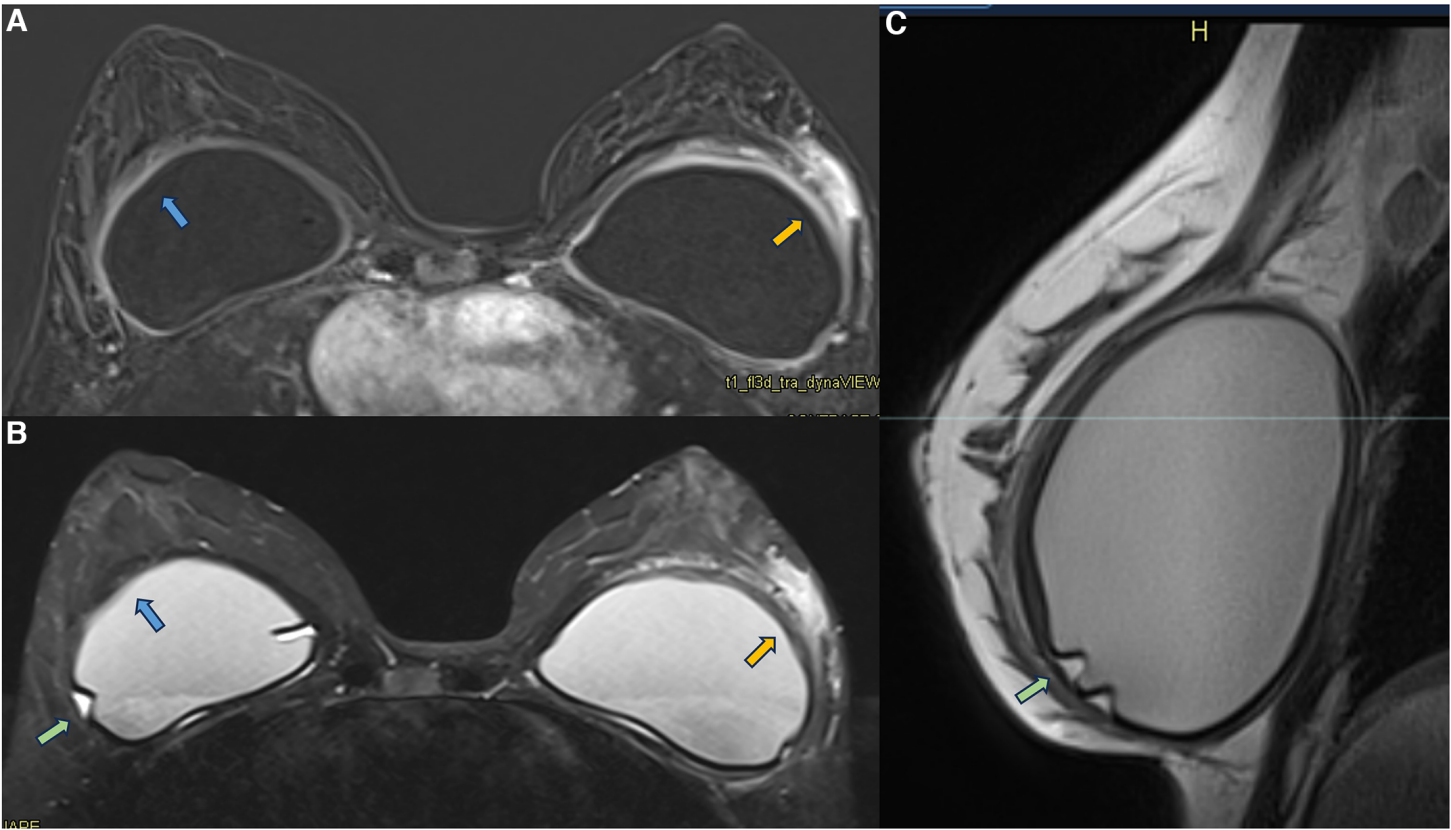
A 36-year-old woman with acute left breast swelling with retropectoral implants for 7 years. The axial post-contrast image (**A**), T2** sequence (**B**), and sagittal proton density sequence (**C**) show a loss of the signal homogeneity of the silicone implants associated with intracapsular granulomas (green arrow). The blue arrows show a moderate inflammatory process of the right fibrous capsule in contiguity with the pectoral muscle (blue arrow). The orange arrow shows inflammatory signs of the left fibrous capsule associated with non-mass enhancement of the pericapsular tissue. Thickening of the pectoral muscle is also observed. The biopsy shows mastitis.

In some cases of patients with symptomatic capsular contracture, who present with clinical signs of breast inflammation, magnetic resonance imaging may show non-mass enhancement of the pericapsular glandular tissue. Non-mass enhancement is a suspicious MRI descriptor finding that should be further investigated. As the foreign body triggers the inflammatory process in the implant fibrous capsule, and due to the evidence of extracapsular migration of silicone particles, there is expected to be an immune reaction in all places with free silicone particles (21, 31, 30, 47) (Figure 9).

Generally, the inflammatory process is self-limited, lasting for about 5 weeks and peaking in intensity in the first 2–3 weeks. Reactive mastitis is the central diagnostic hypothesis, but a differential diagnosis must be employed, and the patient should be forward to a diagnostic biopsy. In these cases, as the differential would be breast carcinoma. These lesions should be classified into category 4 according to the BI-RADS lexicon (lesions suspicious for breast carcinoma).

**Figure 10:**
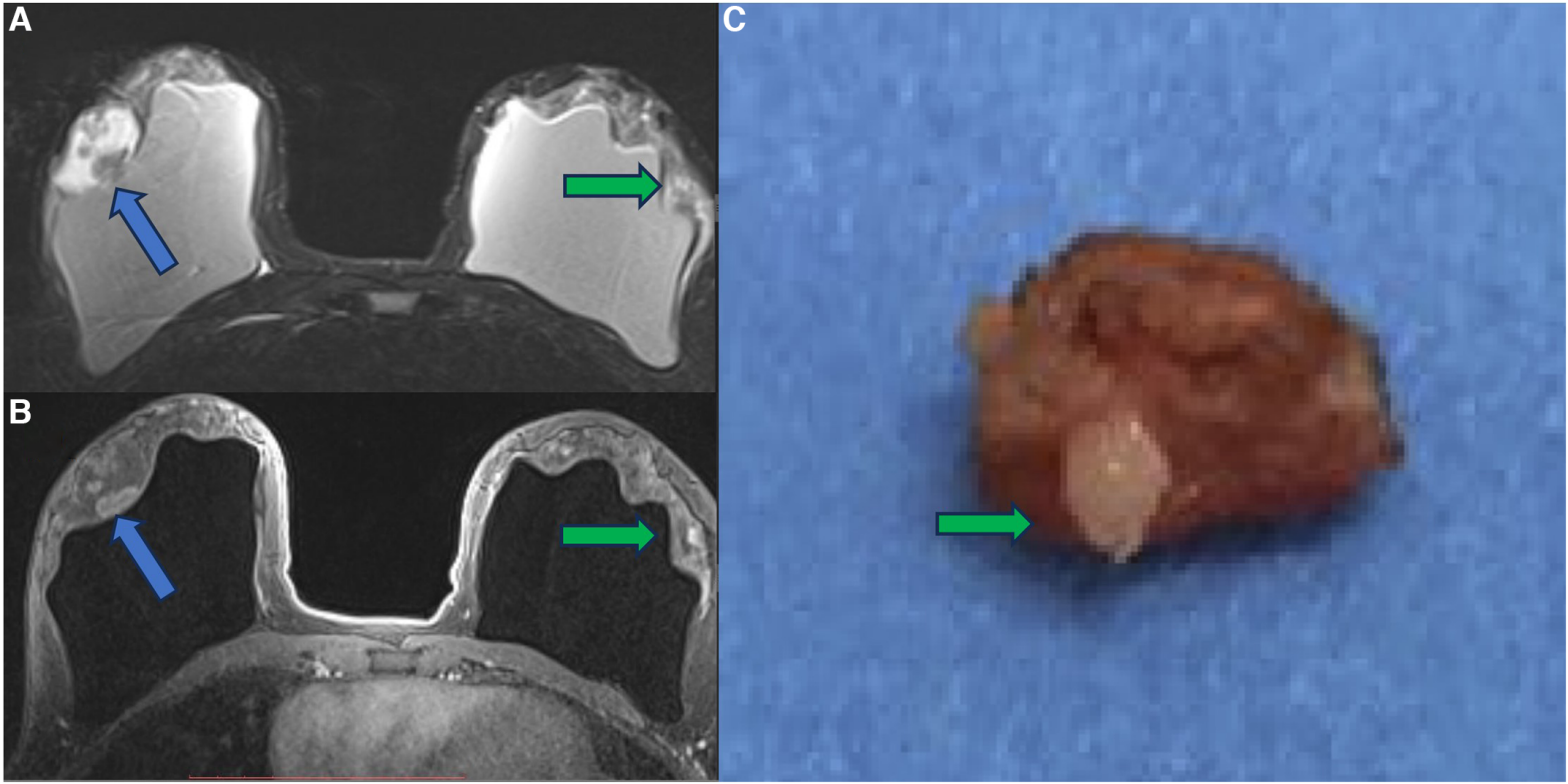
A 41-year-old woman with esthetic breast augmentation with silicone for six years, presenting a mass in the right breast. The axial T2** sequence (**A**) and axial post-contrast (**B**) show a histologically confirmed papilloma in the right breast (blue arrow) and a focal non-mass enhancement on the left breast (green arrow). The specimen of the left breast shows a granuloma with silicone cohesive gel content (green arrow). The imaging integrity of the silicone implants and SIGBIC findings in the left breast MRI imaging are also noted (yellow arrow).

**Figure 11:**
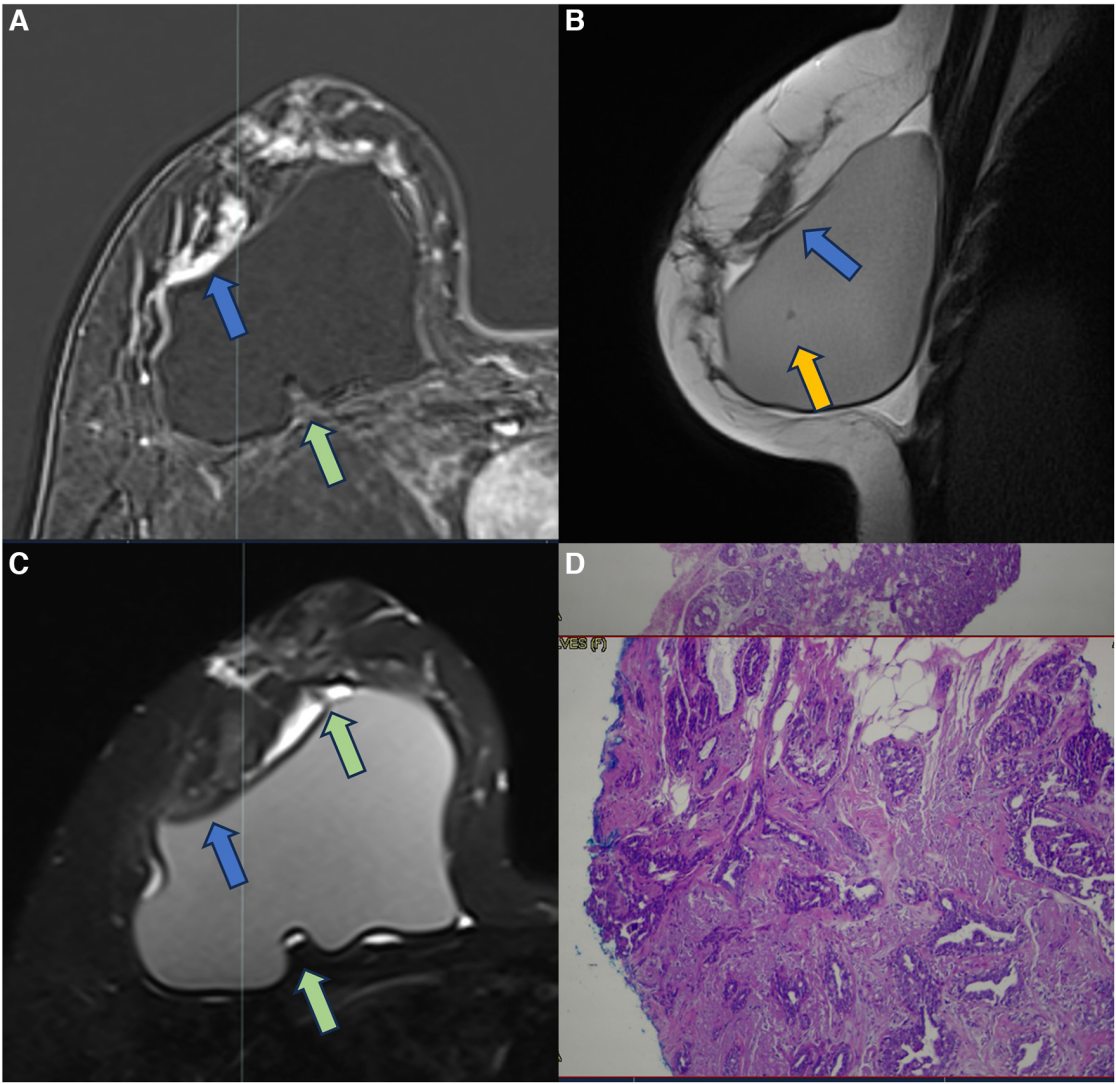
A 37-year-old woman with breast implants for 6 years, presenting capsular contracture of the right breast. The axial post-contrast image (**A**), sagittal proton density image (**B**), and axial T2** sequence (**C**) show a rotated implant with anteriorization of the posterior seal, associated with the fluid collection and SIGBIC (green arrow) and non-mass enhancement of the pericapsular tissue (blue arrow). The water-droplet signal (orange arrow) inferring permeability change of implant surface is also observed. The biopsy specimen shows atypical ductal hyperplasia at the site of the posterior seal.

**Figure 12:**
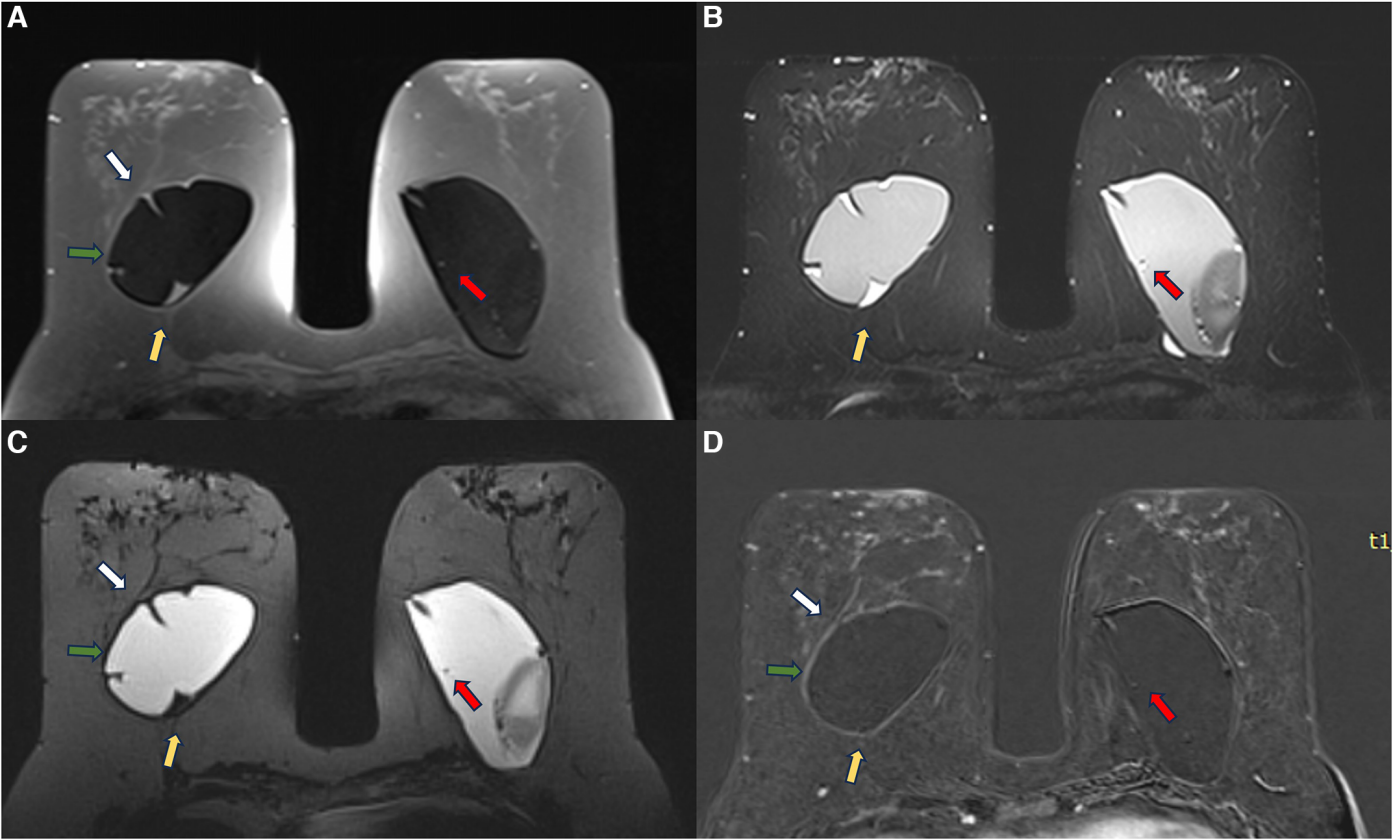
A 53-year-old woman with esthetic retroglandular silicone for 10 years. Preoperative exam done for implant replacement after Baker IV capsular contracture. Axial T1 pre-contrast image (**A**), axial T2** (**B**), axial silicone sensitive sequence (**C**), and axial post-contrast (**D**) showed bilateral capsular contracture associated with SIGBIC (yellow arrow) and intracapsular collection. There is pericapsular edema of the right breast tissue (green arrow) with a black-drop signal and non-mass enhancement of the pericapsular tissue. A water-droplet signal inferring surface permeability change is also noted in the left breast. A ductal carcinoma *in situ* in the right breast was observed at the capsular histology associated with an intracapsular rupture of the breast implant that was not seen in the MRI.

In a case report, we show a patient with a papillary breast lesion developed in the implant shell discontinuity area, where there was direct exposure of the silicone to the breast tissue. This case report corroborated our theory that the chronic inflammatory process of breast implants and silicone particle toxicity could be a triggering factor for metaplasia/dysplasia, ranging from polyclonal benign cells to monoclonal undifferentiated carcinomas (31) (Figures 10–12).

**Figure 13:**
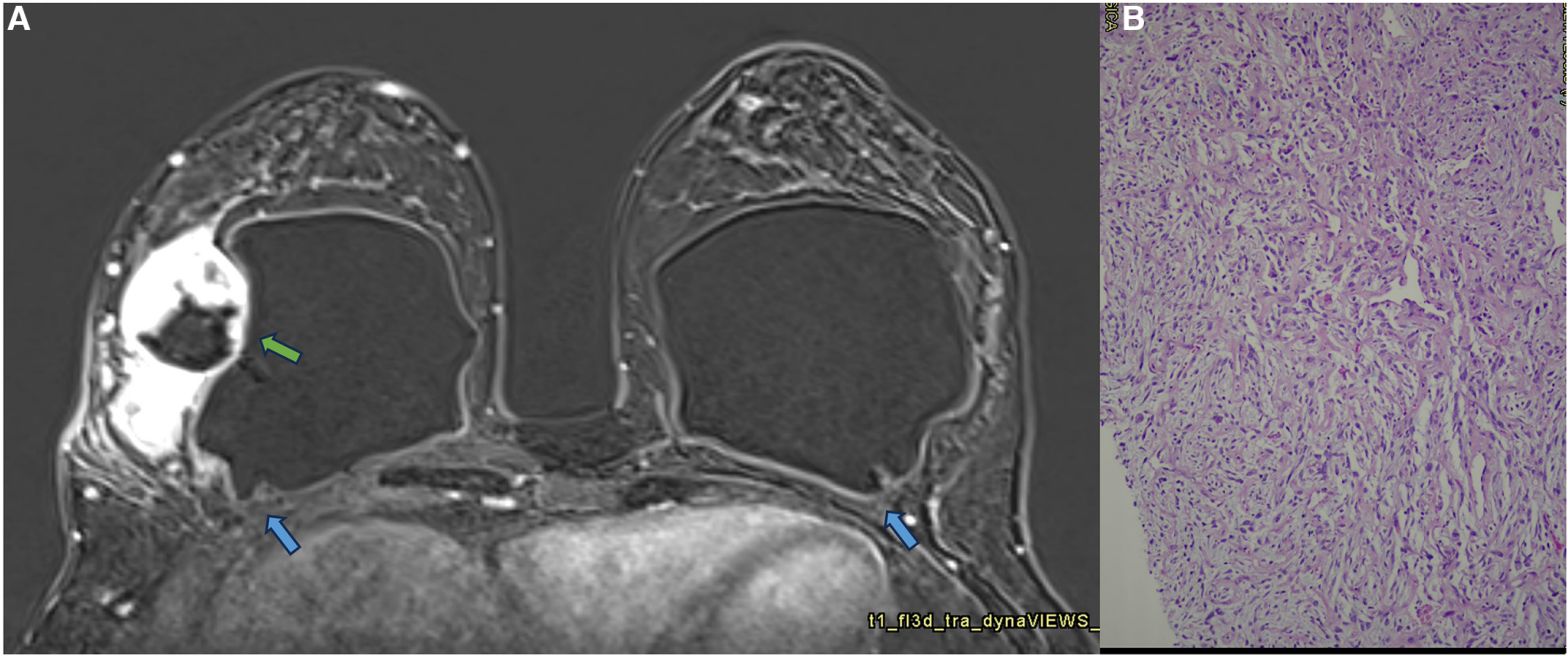
A 42-year-old woman with a developing mass in the right breast. The axial post-contrast MRI image (**A**) shows a pericapsular mass adhered to the fibrous capsule in an area of implant discontinuity (green arrow). SIGBIC is also observed in both fibrous capsules (blue arrow). The biopsy specimen shows a high undifferentiated carcinoma (**B**).

Since we started our study protocol, we still found some rare cases of undifferentiated carcinoma adhered to the fibrous capsule in our clinical practice in patients who were not included in the research protocol (Figure 13). In all the cases, the magnetic resonance showed signs of silicone degradation, SIGBIC, capsular contracture, and a pericapsular inflammatory process. Again, due to the suspicion of breast carcinoma, these lesions should be considered category 4 according to the BI-RADS lexicon.

The FDA recently recognized BIA-SCC as a rare but aggressive malignancy originating from the breast implant capsule. The origin of BIA-SCC is still being determined. It is proposed that ductal epithelium can be displaced at the time of pocket implantation, resulting in squamous epithelialization of the breast implant capsule. According to our experience, the theory of implantation of epithelial cells in the fibrous capsule with subsequent dysplasia when exposed to the inflammatory process is controversial. This lesion's origin would be the contiguity of the inflamed fibrous capsule with the pericapsular glandular tissue. During our study, we found three cases of ductal carcinoma *in situ* (DCIS) in the fibrous capsules of the implants as an incidental finding in patients who underwent explant surgery. The DCIS diagnosis confined to the implant capsule corroborates to our hypothesis (4).

### 6.4. Skin

**Figure 14:**
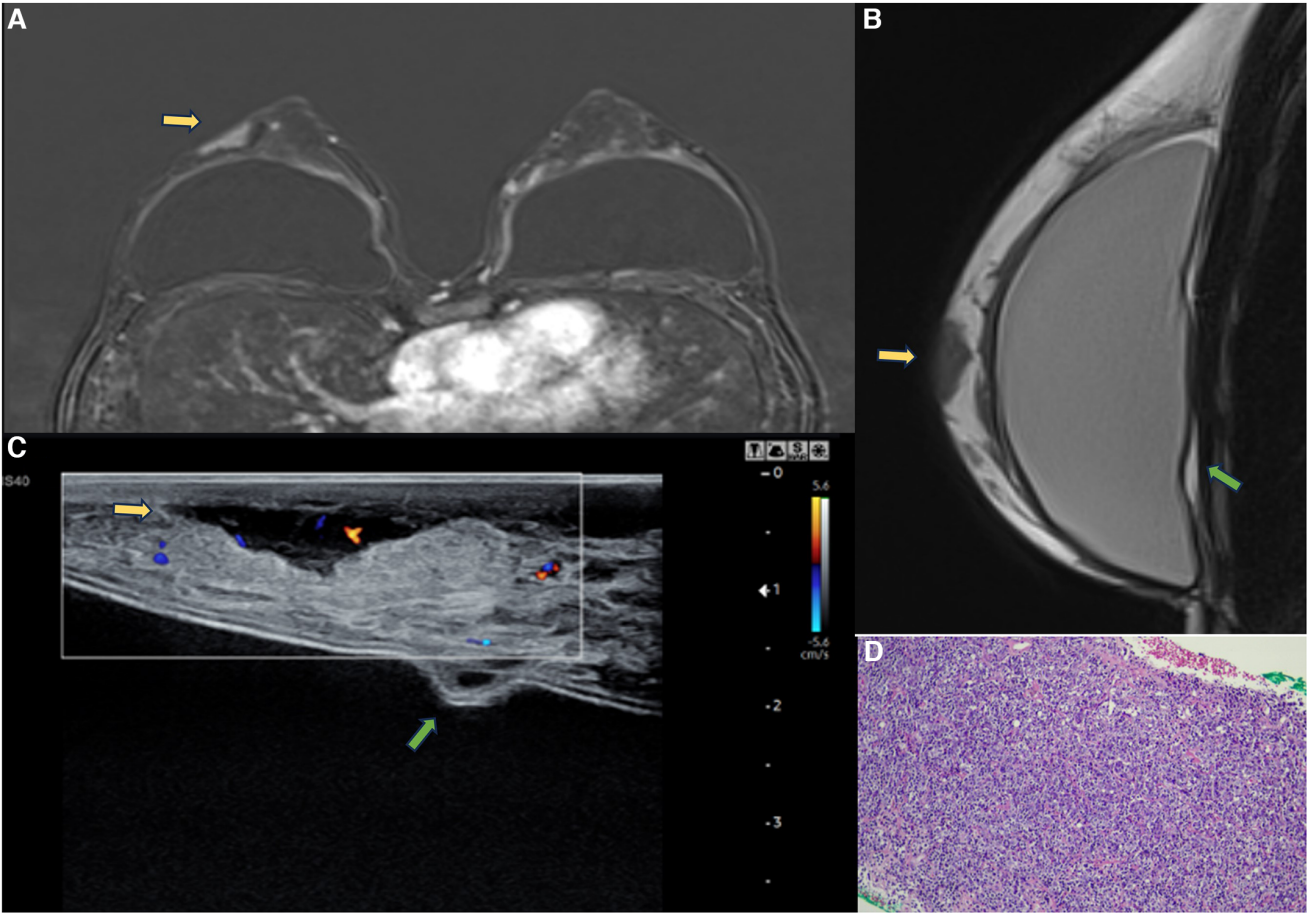
A 47-year-old woman with a breast palpable lump in the right breast. The axial post-contrast MRI image (**A**), sagittal proton density (**B**), and ultrasonography (**C**) show a mass involving the skin and subcutaneous tissue with moderate enhancement (yellow arrows). There is also capsular contracture and SIGBIC (green arrow). The biopsy specimen (**D**) shows epithelioid neoplasia with areas of myoepithelial pattern.

**Figure 15:**
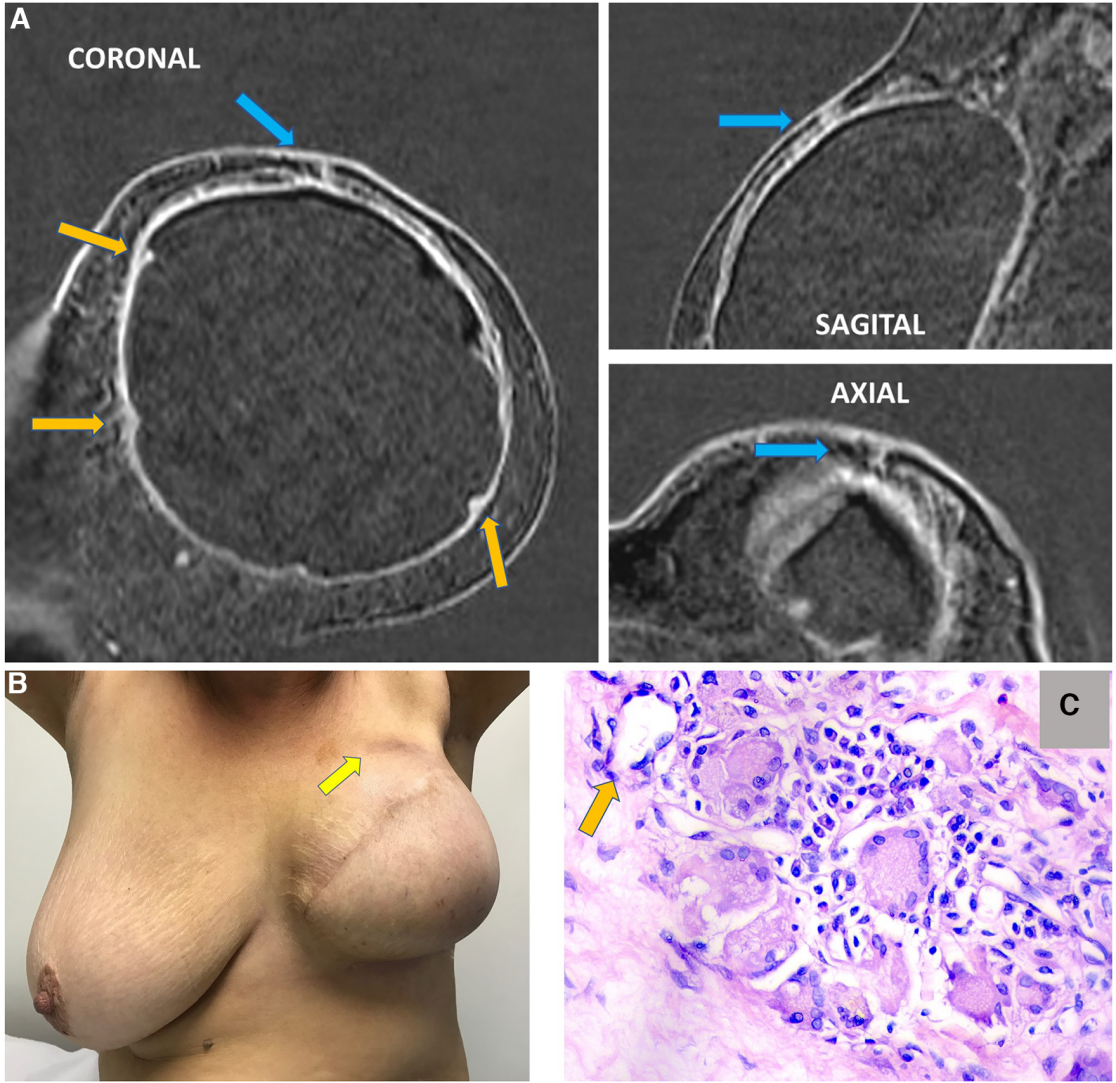
A 63-year-old woman after mastectomy and breast reconstruction with retropectoral silicone implants for 4 years. The patient refers an erythema in the reconstructed breast. The coronal sagittal and axial post-contrast multiplane reconstructed images (**A**) show skin involvement in the inflammatory process in the left breast (blue arrow). There is also capsular contracture and SIGBIC (orange arrows). The pectoral muscle is atrophic in contiguity to the fibrous capsule. (**B**) shows skin erythema in the site of the imaging findings. (**C**) demonstrates the biopsy specimens with a silicone-induced granuloma (orange arrow shows a giant cell with free silicone corpuscle).

The fibrous capsule inflammatory process can extend to the skin and subcutaneous tissue depending on the proximity of the implant to the subcutaneous skin, especially in patients undergoing oncological surgeries. In addition to the inflammatory process related to capsular contracture, silicone corpuscles may migrate to the skin and subcutaneous tissue. In these cases, erythematous lesions on the skin are seen in the presence of an acute inflammatory process of the fibrous capsule. In some cases, the inflammatory process can extend to the surgical scar (Figures 14, 15).

In magnetic resonance images, it is possible to determine the contiguity of the fibrous capsule with the skin and subcutaneous tissue. Magnetic resonance imaging often shows irregularities and changes in the signal of the implant in the topography of the skin lesion. Describing the skin involvement in the imaging scan for surgical planning is imperative to remove the diseased tissue in a new surgical approach.

## 7. Conclusion

Since the middle of the 2010 decade, the debate regarding silicone implant safety has been in evidence. From concepts that cohesive gels were inert and biocompatible to evidence of breast implant illness, BIA-ALCL, and BIA-SCC, silicone implants evoke a contradictory debate. However, little attention is paid to the impairment of the pericapsular space.

Many silicone implant complications are underdiagnosed because of the lack of silicone-related diseases knowledge, study protocols standardization, and dedicated interpretation guidance to screen and diagnose the diseases related to these devices. Imaging findings related to silicone implants should be interpreted and classified independently of the BI-RADS lexicon.
